# Influence of rice husk ash on the mechanical properties of ultra-high strength engineered cementitious composites (UHS-ECC)

**DOI:** 10.1371/journal.pone.0301927

**Published:** 2024-04-18

**Authors:** Feifei Liu, Baohong Jin, Qi He, Yun Zhou

**Affiliations:** 1 Architectural Engineering Institute, Zhejiang Tongji Vocational College of Science and Technology, Hangzhou, China; 2 School of Civil and Hydraulic Engineering, Ningxia University, Yinchuan, China; SASTRA Deemed University, INDIA

## Abstract

Generally, UHS-ECC should consume massive cement, which is negative to its sustainability as cement production leads to 8% of global CO_2_ emissions. To decrease the cost of production and carbon emissions of UHS-ECC, rice husk ash was employed to replace the cement as a supplementary cementitious material in this study. Experiment results illustrate that blending rice husk ash (RHA) would decrease the fluidity of mortar. Furthermore, the green UHS-ECC shows a maximum compressive strength of 130.3 MPa at 28 days when RHA content was 20% of cement. The ultimate tensile strength of UHS-ECCs first increased and then decreased, while both tensile strain and strain energy presented an opposite tendency. At the micro-scale, if RHA content was lower than 20% of cement, incorporating RHA can significantly decreasing fiber bridging complementary energy of UHS-ECC, thus reducing pseudo strain hardening energy (PSHenergy) index, which finely agrees with the degradation of ductility of UHS-ECCs. To guarantee the features of ultra-high strength, acceptable workability, and high tensile ductility, the RHA dosage should not be in excess 20% of cement. These researched results are prospected to the contribution of pozzolanic RHA on the efficient usage of sustainable UHS-ECC.

## 1. Introduction

Brittleness and vulnerability are the ineluctable weaknesses of traditional concrete in the worldwide infrastructure, especially when these superstructures are subjected to hypernomic load, thus leading to catastrophic destruction. High-strength concrete is usually along with more brittle [1]. To date, ultra-high-performance concrete (UHPC) with high flexibility and ultra-high-strength has been employed in many structures such as bridge decks, piers, and tunnel walls [2, 3]. However, a single thick crack emerged when UHPC fractured due to its low ductility, which inducted external erosion solution into the interior matrix and reduced the durability of the UHPC structure [4]. Hence, the special cementitious materials with high strength, high toughness, and high ductility urgently need to be developed to enhance the matrix crack resistance ability.

Engineered cementitious composites (ECC) were designed by the micromechanical code and fracture mechanics, which can achieve pseudo-strain hardening properties and show many tiny cracks under the tensile process [5]. Additionally, it usually possesses tensile ductility at a range of 3–8%, which has a popular compressive stress of 20–80 MPa [6, 7]. To pursue mair strength and ductility for reinforced concrete structures, the ultra-high strength ECC which possesses a ductility of exceeding 3% and a strength of over 120 MPa was successfully fabricated in laboratory experiments, which usually blended polyethylene (PE) fiber or hybrid PE and short fine steel fiber [8–11]. To date, Huang et al. [12] has successfully manufactured the UHS-ECC which shows a ductility of 6.3% and a largest compressive stress of 211 MPa based on the energy-based criterion and strength-based criterion of ECC. However, a low sand/binder ratio in a range of 0.25–0.30 was usually employed to obtain a reasonable matrix fracture toughness and achieve multiple cracking based on the design guideline of ECC [13, 14]. Hence, the cement content in the UHS-ECC was usually over 1000 kg/m^3^ which is higher than around 3–4 times that of popular concrete. Generally, the production of per 1kg cement will release 0.87 kg carbon [15, 16]. Therefore, it is urgent to develop a green UHP-ECC that some potential supplementary cementitious materials (SCMs) or low-carbon cement is usually employed to substitute part of the cement, thus impairing the amount of carbon oxide emissions of UHS-ECC, which is consistent with the green sustainable development strategy of China. Lao et al. [17] reported that geopolymer composites could be employed to produce the high strength engineered geopolymer composites with a compressive strength of over 140 MPa and high tensile ductility of around 8%. Moreover, Zhu et al. [18] found that magnesium oxysulfate cement could be used to prepare high strength PE-ECC with green, lightweight, thermal insulation and hydrophobic functions. Although geopolymer composites and magnesium oxysulfate cement show a low carbon property compared to traditional Portland cement, their rapid coagulation defect makes relative ECC difficult to apply on-site. Hence, the available industrial by-products such as limestone powder, slag, and fly ash were usually applied to decrease the cement dosage in ECC mixtures [19–21]., while above industrial by-products continuously decrease under the green sustainable development strategy of China, thus developing new SCMs can decrease the carbon emission of UHS-ECC and meet the green concrete industry development.

Rice husk is an agricultural by-product, in which 20% of the grain is husk, and approximately 20% of the husk is converted to ash after combustion [22]. In 2018, the global rice husk took up around 160 million tons of weight, while massive rice husk is relegated to landfills, which does not maximize utilize available resources [23, 24]. After burning under 700°C, RHA possesses massive amorphous SiO_2_, which exhibits a great pozzolanic activity and filling effect. The high pozzolanic activity, that is, autologous noncrystalline silica can originate a reaction with portlandite to manufacture the new low Ca/Si C-S-H gel [25]; moreover, RHA possessing a foraminate structure can provide the nucleation point for hydration products, thus effectively boosting the matrix strength and durability [26]. Based on the merit of pozzolanic RHA, it is more reasonable to use pozzolanic RHA as a substitute for cement in UHS-ECC. To date, few studies focused on investigating the usage of pozzolanic RHA in ECC preparation. Da Costa first reported that the non-processed rice husk ash substitute 30% of cement (by volume) could reduce free and bring shrinkage of ECC [27]. Additionally, Yaswanth found that RHA could replace partial ground granulated blastfurnace slag (GGBS) to produce engineered geopolymer composites as it has a high pozzolanic property [28]. Zhang designed a high strength ECC mixture with RHA, and found that RHA could lower the matrix toughness of ECC and increase the PSH index However, the used RHA in the above references exhibited a black color that amount of carton was presented in black RHA, which could not give full play to its pozzolanic effect, and also impacted the tensile characteristics of ECC since the fiber/matrix interfacial frictional bond is closely related to the stiffness and compactness of the interfacial transition zone (ITZ). Set against this background, there are no reports focusing on the effect of pozzolanic RHA on the comprehensive performance of UHS-ECC.

This study aims to produce a green ultra-high strength ECC by substituting cement with pozzolanic RHA. It is noteworthy that the used pozzolanic RHA burned under 800°C possesses a great high activity, which is different from the traditional RHA that could not employed as an SCM due to its high carbon content and low noncrystalline silica content considering the hydroscopicity of RHA, the fluidity of the paste was conducted to select a suitable workability of UHS-ECC. Besides that, the mechanical properties of UHS-ECCs containing RHA were explored. The phase components and quantity of hydrates in the mortar with RHA were measured via X-ray diffraction (XRD) and thermogravimetric (TG) analysis, correspondingly. Based on the strength criteria and energy design criteria of conventional ECC, the ductility of UHS-ECC containing pozzolanic RHA was interpreted. Three-point bending test was tested to calculate the fracture toughness of the mortar, while a single crack test was implemented to gain the fiber bridging strength to crack opening width correlation. Finally, the microstructure of mortar containing RHA was measured by the scanning electron microscope (SEM) analysis. Importantly, the experiment results are desired to offer references for the recycling of agricultural rice husk as a pozzolanic SCM, which also reduces the cement dosage and makes UHS-ECC more eco-friendly.

## 2. Experimental investigations

### 2.1 Materials

In this work, Ordinary Portland cement (P·O 52.5), pozzolanic rice husk ash, silica fume (SF), high-modulus polyethylene (PE) fiber, superplasticizer (SP), silica sand, and water were applied to prepare UHS-ECC. The RHA was obtained after the rice husk was burned under 800°C for 72 h in the burning oven, which was provided by a rice plant in Maoming (Guangdong province, China) Table 1 lists the chemical compositions of binders. The grey silica fume was employed to enhance the matrix strength due to its superior pozzolanic activity, which contains 96% SiO_2_. The specific gravity of RHA is 2.2 g/cm^3^, while its specific surface is 65 m^2^/g. The silica oxide of pozzolanic RHA was around 88%, slightly lower than that of SF. Table 2 illustrates the physical and geometric parameters of PE fiber. The PE fiber with a density of 0.97 g/cm^3^ has a length of 18 mm and 26 μm in diameter. The fiber nominal tensile strength and elastic modulus are 3000 MPa and 100 GPa, respectively. Polycarboxylate superplasticizer was produced by Sika Group, which has a water decrement capability of around 40% and can reach the desired flowability of the paste.

**Table 1:** Chemical properties of binders (wt. %).

	SiO_2_	Al_2_O_3_	Fe_2_O_3_	CaO	Na_2_O	MgO	K_2_O	MnO	SO_3_	LOI
**Cement**	21.3	5.42	3.46	61.8	0.61	2.90	0.60	-	1.74	2.17
**RHA**	88.16	0.21	0.22	1.35	0.00	0.80	4.60	0.70	0.39	3.59
**SF**	96.90	0.30	0.15	1.54	0.16	0.18	0.64	-	-	0.03

LOI: loss on ignition

**Table 2:** The physical properties and geometrical dimensions of polyethylene fiber.

Elastic modulus /GPa	Tensile stress/MPa	Diameter /μm	Length /mm	Density /g/cm^3^
100	3000	24	18	0.97

**Fig 1:**
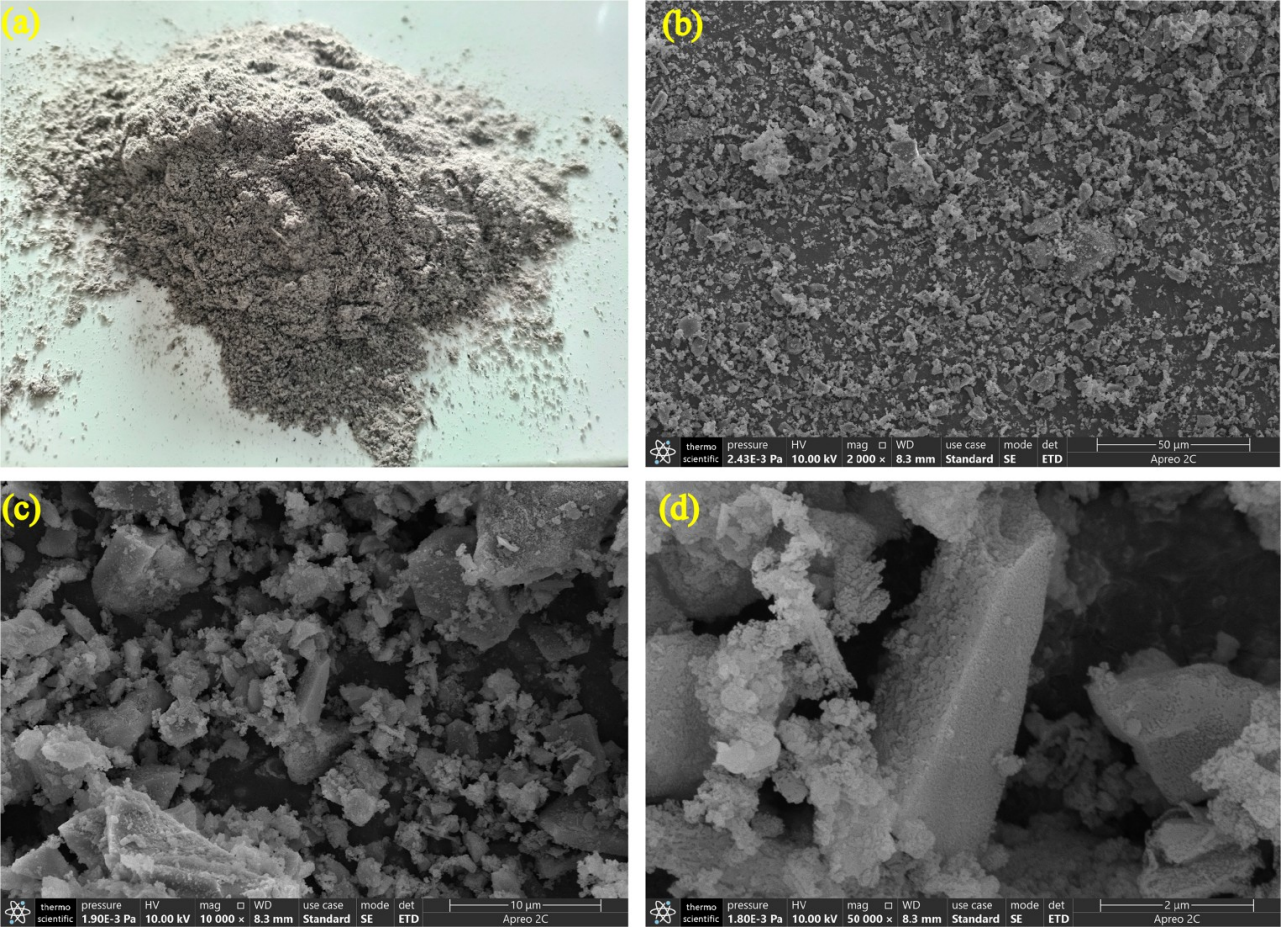
(a) macrograph and micrograph images of RHA before ball milling: (b) 2000×, (c) 10000 ×, and (d) 50000×.

**Fig 2:**
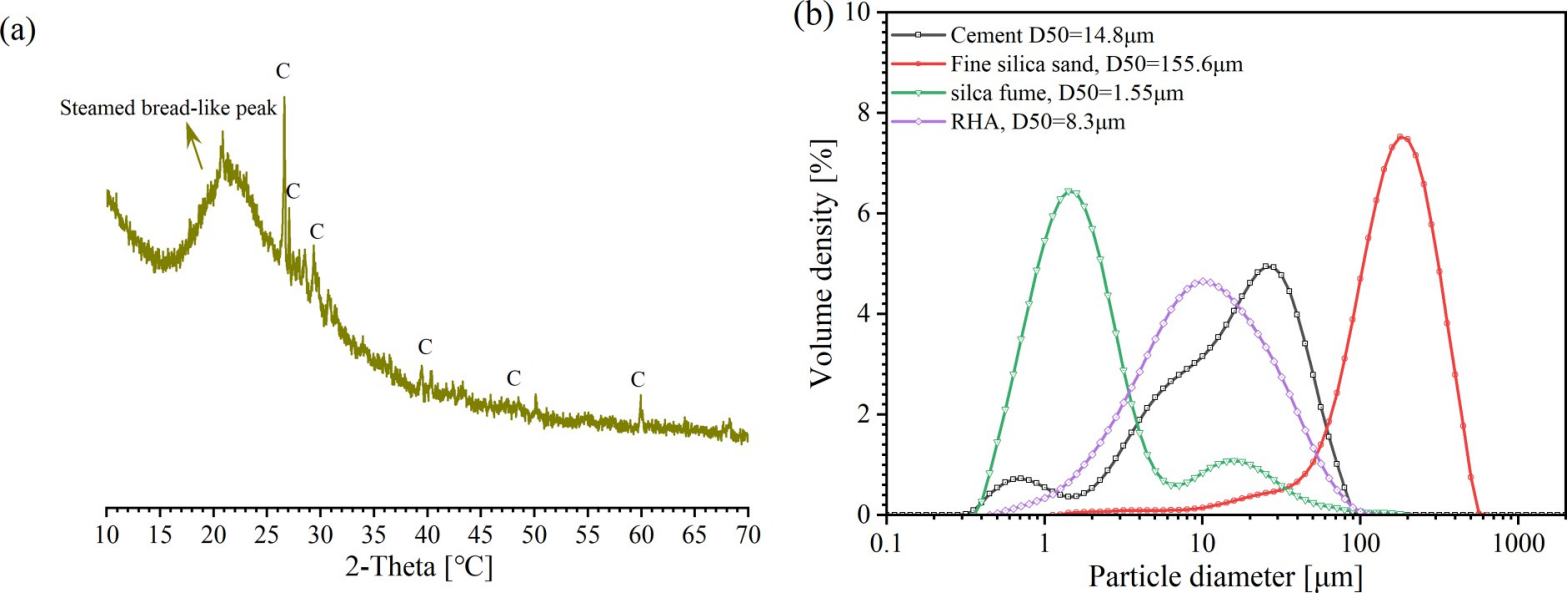
(a) XRD phase components of RHA (C: cristobalite quartz); (b) particle distribution of solid materials.

Fig 2 a shows the XRD phase components of RHA, which has a steamed bread-like peak ranging from 10° to 30° in the 2θ angles, indicating that RHA has much amorphous silica. In addition, the XRD Rietveld analysis found that the crystallinity of SiO_2_ was only 11.93%, indicating that few cristobalite quartz was also presented in RHA since amorphous silica could be transformed into crystalline when the burning temperature exceeds 700°C[29]. In addition, the morphology and microstructure of ground RHA are observed in Fig 1, where the ground RHA has an irregular angular structure, and shows massive porous particles on the surface of RHA (see Fig 1D).

A laser particle sizer was applied to measure the particle size distributions of solid composites of UHS-ECC, as shown in Fig 2B. The D_50_ of cement, SF, and RHA were 14.8 μm, 1.55 μm, and 8.3μm, respectively. It is noteworthy that a low particle size of rice husk ash has a low hydrophilia since many pore structures of RHA particles were destroyed in the ground process [30]; moreover, a finer particle of RHA shows a higher pozzolanic activity. Owing to the design criteria of popular ECC, silica sand was usually employed to reduce the matrix toughness; thus, a fine average grain size of 155.6 μm for silica sand was employed in this study.

### 2.2 Mix proportions and preparation process of UHS-ECCs

Table 3 demonstrates the mixture ratio of UHS-ECCs. Owing to the researched findings, the concrete strength of the mortar with RHA could be improved if RHA content was lower than 20% of cement [31]. Thus, in UHS-ECC (M-10, M-20, M-30), RHA replaced 10%, 20%, and 30% of cement by mass, respectively. The sand/biners and water/binders are 0.42 and 0.19, correspondingly. Meanwhile, a SP-to-binders of 1.7% was applied to maintain a suitable flow performance of UHS-ECCs. Moreover, to ensure ductility, the volume of PE fiber was selected as 2%.

**Table 3:** Mixture ratio of UHS-ECCs (kg/m^3^).

Number	Cement	RHA	SF	Silica sand	Water	SP	PE fiber (by volume)
**M-C**	1400	0	250	693	304	40	2%
**M-10**	1260	140	250	693	304	40	2%
**M-20**	1120	280	250	693	304	40	2%
**M-30**	980	420	250	693	304	40	2%

Firstly, the solid materials were dumped into a mortar mixer and stirred for 3 min. Then, all water and SP solution are poured and stirred for 2 min. Finally, throw PE fibers into the fresh paste until all fibers were well dispersed, which will consume 10 min during this period. Afterthat, the liquid paste was poured into prepared moulds and overspread plastic film until demoulding after 24 h. All samples were placed in the relative humidity of 90 ± 5% and temperature of around 25°C for 28 days.

### 2.3 Experimental methods

#### 2.3.1 workability

The fresh property of UHS-ECC mortar was tested by the flow table per ASTM 1437 [32]. All fresh composite spread after 25 blows, then the largest diameter at two orthogonalization orientations was used to calculate the average spread diameter of the mortar.

#### 2.3.2 X-ray diffraction analyses

XRD analysis was conducted to explore the phase components of hydrates. After curing for 28 days, the hardened mortar was invaded into alcohol to stop the hydration process. In addition, all mortar fragments were dried in the oven for 120 min at 50°C. Thereafter, grinding the dried pastes into powders to pass the sieve and gain the pure pastes with no nonreactive silica sand. After that, all powders were set on an analyzer (Rigaku Ultima IV, Japan), and the scanning ranging from 5° to 85° (2θ) with a theta increment of 0.02°/step and was conducted in a Cu-Kα irradiation circumstance of 40 kV and 40 mA with a minimum wavelength of 0.0001 degree.

#### 2.3.3 thermogravimetric analyses

To quantitatively obtain the portlandite content in mortar containing RHA, the prepared powder (around 50 mg) was set on the holder and placed on an analyzer (NETZSCH STA 449, Germany). Firstly, the hydration procedure of mortar was ceased by using ethyl alcohol. Whereafter, all mortar fragments were desiccated in an oven for 120 min at 50°C, and grinding the dried pastes into powders to pass the sieve and gain the pure binder products. The heating temperature lies between 40 and 1000°C with 20°C/min in the heating atmosphere of N_2_. Additionally, the derivative thermal gravimetric (DTG) curve could be obtained by the first-order derivation of the TG curve, which was used to calculate the portlandite contents of mortar which could be estimated using the following Eq (1) [33, 34].


$${Ca(OH)}_{2}=\frac{w_{450}-w_{550}}{w_{550}}∙\frac{74}{18}$$ (1)

Where *w*_450_ and *w*_550_ are the mass loss at the temperature of 450°C and 550°C in the DTG curve, respectively.

#### 2.3.4 Mechanical properties

According to GB/T 17671–2021 [35], the compressive test was conducted on three samples having a module size of 40× 40× 160 mm^3^. The compressive strength of mortar could be obtained by averaging these three mortar specimens, and the load rate was 2.4KN/s.

**Fig 3:**
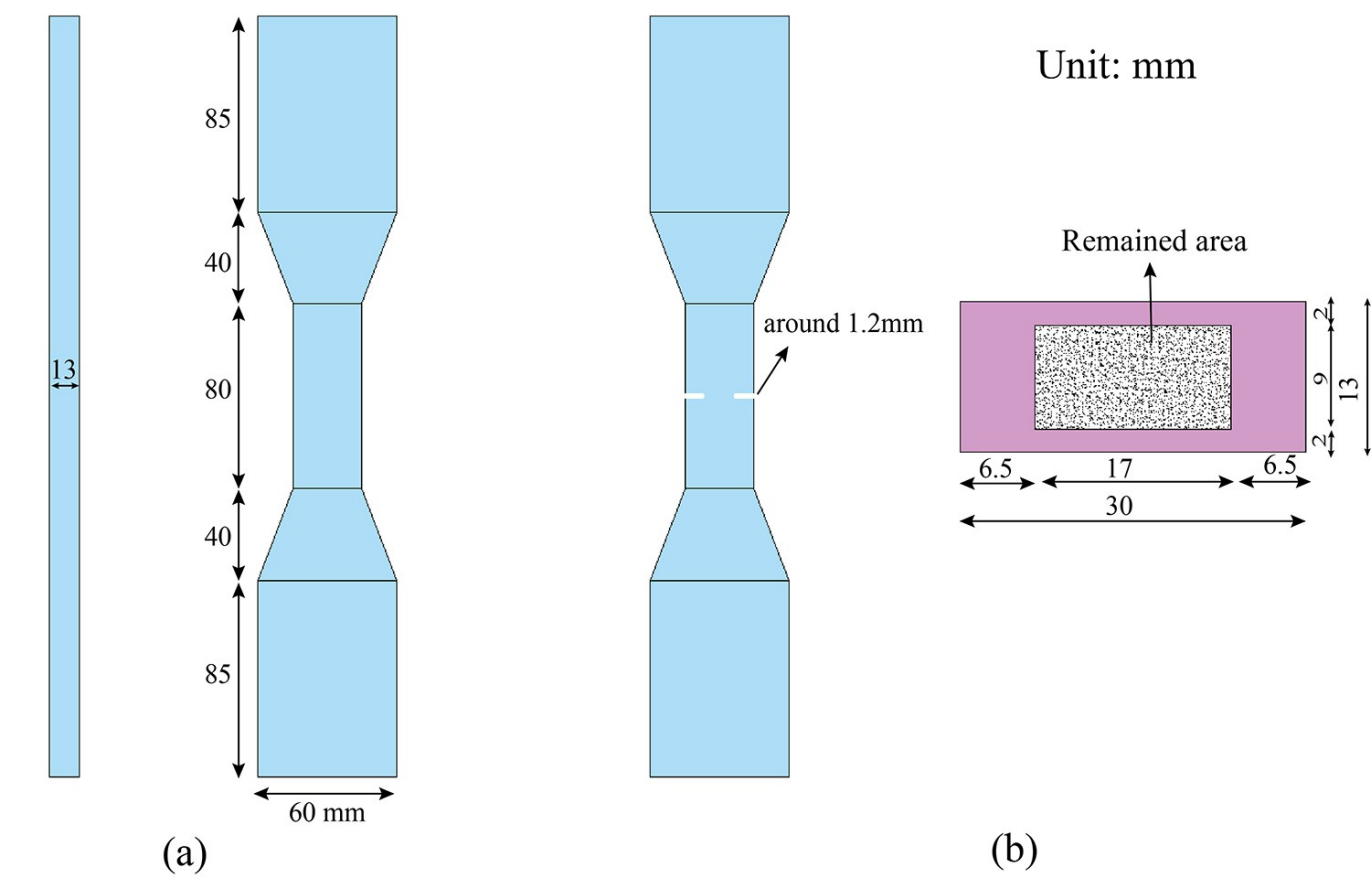
Dog-bone specimens for (a) tensile test and (b)single-crack tension experiment.

Per JSCE-2008 [36], the tensile test was used to explore the tensile characteristics of UHS-ECCs, and the dog-bone specimens are shown in Fig 3A. The length, width, and thickness of the measure area are 80 mm, 30mm, and 13 mm, respectively. During the test, the length deformation of 80 mm in the specimen was captured by an extensometer which clinged to the specimen surface, and then the tensile ductility could be gained. The test was conducted under quasi-static loading status with the loading-rate of 1mm/min.

To capture the fiber bridging stress to crack opening width correlation of ECCs containing RHA, a single crack tensile test was performed in this study. All specimens were cut on four faces to form a single crack, as demonstrated in Fig 3B. A saw blade was used to notch the crack with a width of 1.2 mm. The remained area in the cross section of the tensile specimen is 17 × 9 mm^2^. From the fiber bridging stress to the crack opening width curve, the fiber bridging complementary energy could be calculated. The loading rate of the single crack tensile test was 0.01mm/s.

**Fig 4:**
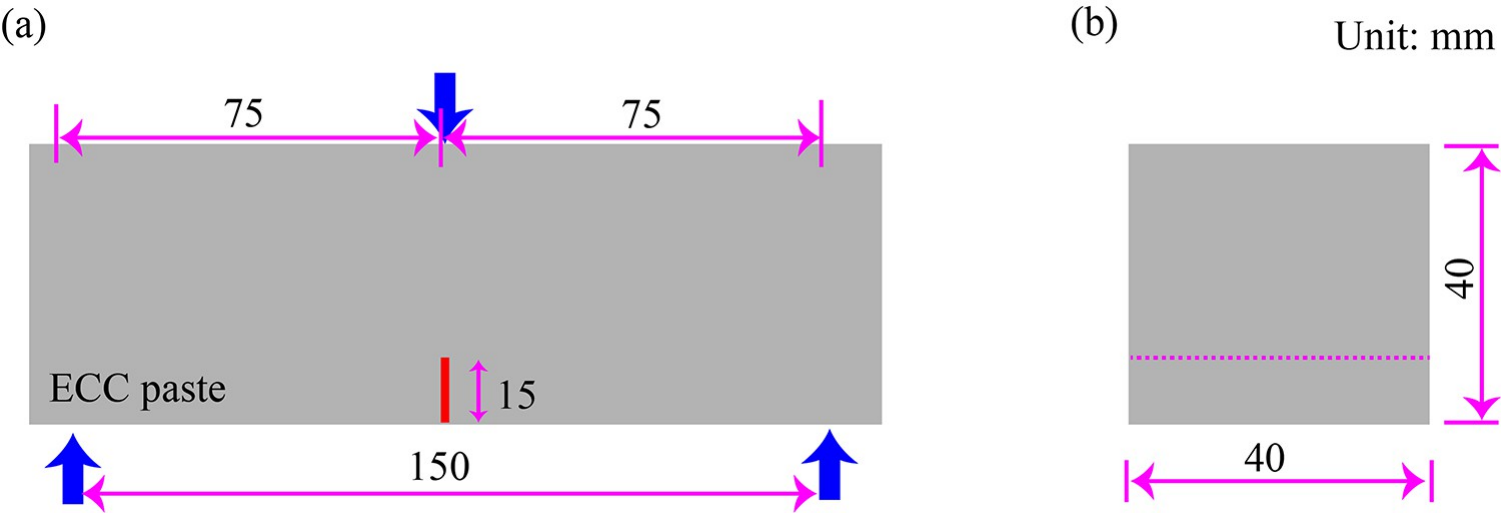
Three-point flexure experiment. (a) front side and (b) transverse elevation.

Three-point flexure experiment of mortar containing no fiber was prepared to research the impact of RHA on the matrix toughness in accordance with ASTM E399 [37]. Hence, beams of 40 × 40 × 160 mm^3^ were cast for each mixture. Three pre-notched specimens were illustrated for each group, whose dimensions are illustrated in Fig 4. The initial notch depth is 15mm, while the loading span is 150 mm with a loading rate of 0.2 mm/min.

#### 2.3.5 Scanning electron microscope (SEM) analysis

For the reference group M-R and M-20 group, the hardened pastes were crushed and invaded in isopropanol to stop the hydration. Afterthat, all pastes were baked in the oven for 120 min at 50°C. Then, three types of sandpaper with 2000 mesh, 1200 mesh, and 800 mesh were applied to ground the hardened fragments; afterthat, gold was used to coat the polished samples. The morphologies of samples were observed by SEM (Thermo Scientific Helios 5 CX, American) attaching an X-ray spectroscopy (EDX) photodetector. Moreover, EDX-pointing was employed to obtain the element composition of UHS-ECC with RHA.

## 3. Results and discussion

### 3.1 Fluidity of UHS-ECC

**Fig 5:**
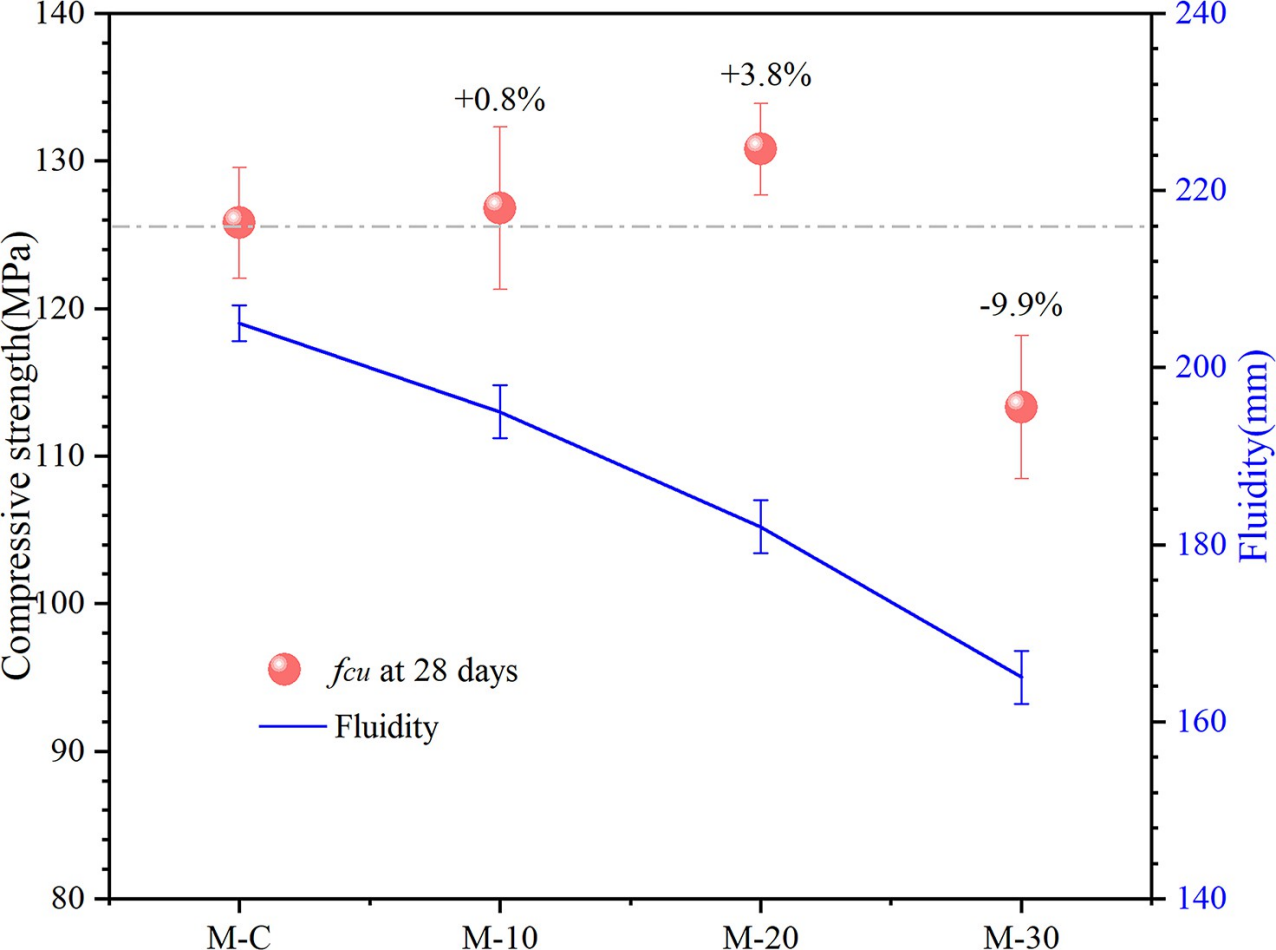
Compressive strength development and fluidity of high strength ECC.

Fig 5 demonstrates the fluidity of UHS-ECCs. It can be seen that the fluidity of M-10, M-20, and M-30 are 195mm,182mm, and 165 mm, respectively, which is lower than 205 mm of M-C. The fluidity of pastes decreases with the increasing RHA dosage, which is likely due to the absorbing water property of RHA possessing a large specific surface of 65 m^2^/g. To date, the fluidity of UHS-ECCs usually ranges from 160 mm to 180 mm [38] since low water to binder and amount of cement was used to achieve the large compressive strength, thus restricting the industrial production of UHS-ECC. Hence, considering the workability of UHS-ECC mortar on foundation engineering, the substitute dosage of cement by RHA should not excess 20% of cement.

### 3.2 Compressive strength development

The compressive strength of UHS-ECCs at 28 days is plotted in Fig 5. From Fig 5, the compressive strength of UHS-ECCs increases first and then mitigates with the increment of RHA dosage. M-10 and M-20 possess a compressive strength of 126.8 MPa and 130.8 MPa, respectively, enhanced by 0.8% and 3.8% compared to the M-C of 125.8 MPa, which could be explained by the pozzolanic reaction of RHA that originate with portlandite to fabricate new C-S-H gel, thus improving the mortar strength [39], which would be discussion in section 3.6. Moreover, the filling effect of pozzolanic RHA with a fine porous structure could provide the hydration nucleation point to contribute to the hydration process, making the interfacial transition zone of mortar with RHA denser than usual mortar [31].On contrary, M-30 has the lowest compressive strength since excessive replacement of cement by RHA would lead to an inadequate hydration product in a mortar. Hence, the maximum compressive strength of mortar could be achieved if RHA replace around 20% of cement, which shows an approximate conclusion in previous research [40, 41].

### 3.3 XRD analysis

**Fig 6:**
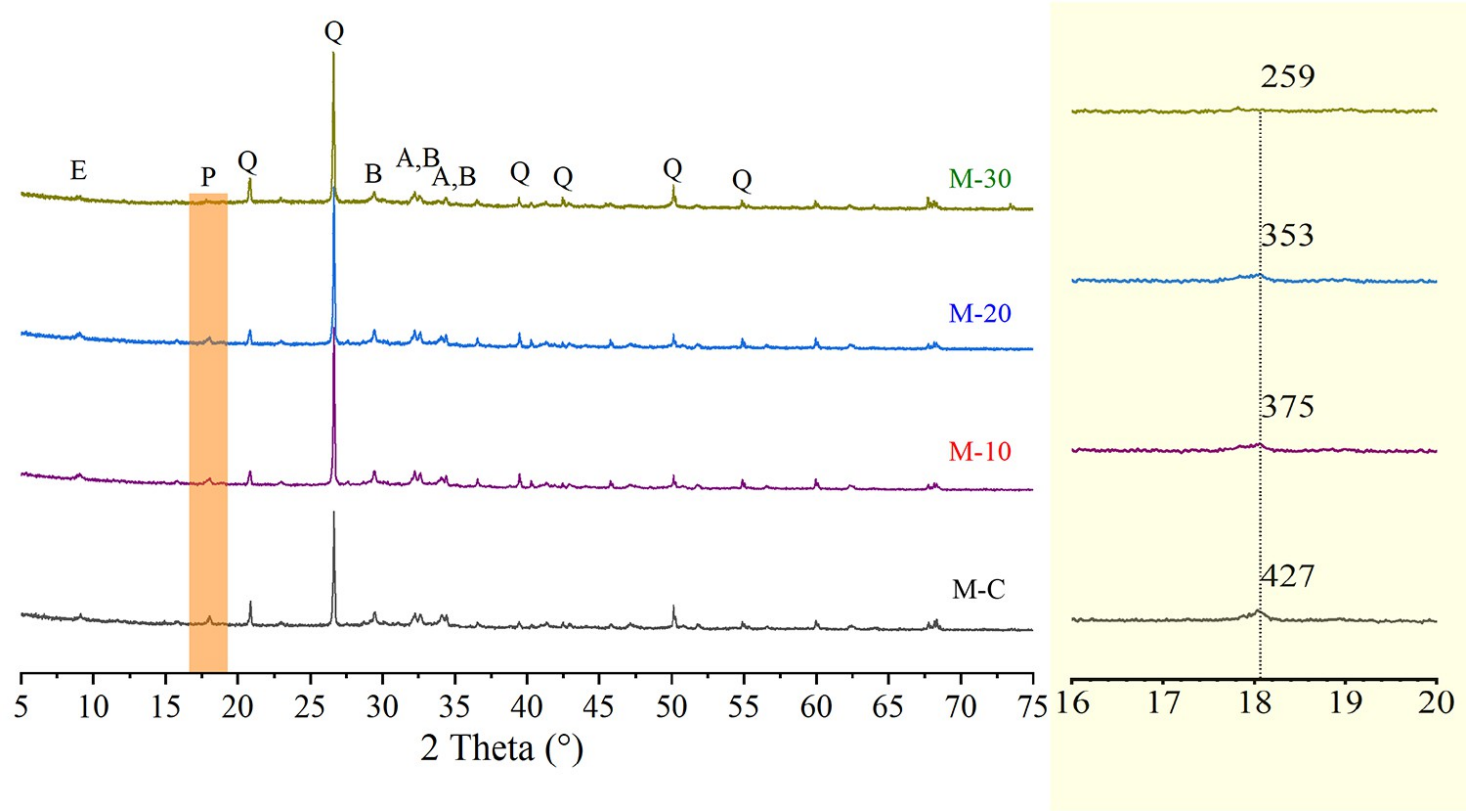
XRD patterns of UHS-ECCs mortar at 28 days. A: Alite-C_3_S; B: Belite-C_2_S; E: Ettringlite-AFt; Q: Quartz-SiO_2_; P: Portlandite-Ca(OH)_2_.

The results of XRD pattern of UHS-ECCs are demonstrated in Fig 6. The Portland cement clinkers containing alite, belite, ettringite, portlandite, and quartz could be found in the XRD analysis. The porous pozzolanic RHA can absorb free water due to its large specific surface area, leading to the incomplete hydration of partial cement; thus, all UHS-ECC mortar has little unreacted cement [42].

From Fig 6, the peak-intensity value of portlandite (2θ≈18.0°) decreases gradually with the increasing replacement of pozzolanic RHA. The portlandite was produced after the cement hydration process. Moreover, the portlandite also react with RHA to generate new Ca/Si C-S-H gel that can contribute to gaining the strength for UHS-ECCs. In paste, the decreasing cement led to a few portlandite. Furthermore, RHA also consumes massive portlandite due to its superior pozzolanic reactivity, which also decreases portlandite content in matrix. In addition, incorporating RHA led to strengthening the peak-intensity value of ettringite in M-10 and M-20 (2θ≈9.0°), indicating that blending RHA could contribute to the hydration process of cement to produce more ettringite in matrix that dense the microstructure of mortar to a certain extent. However, the intensity of ettringite in M-30 was largely lower than that of M-C, which is attributed to the reduction of cement in the matrix.

### 3.4 TG analysis

**Fig 7:**
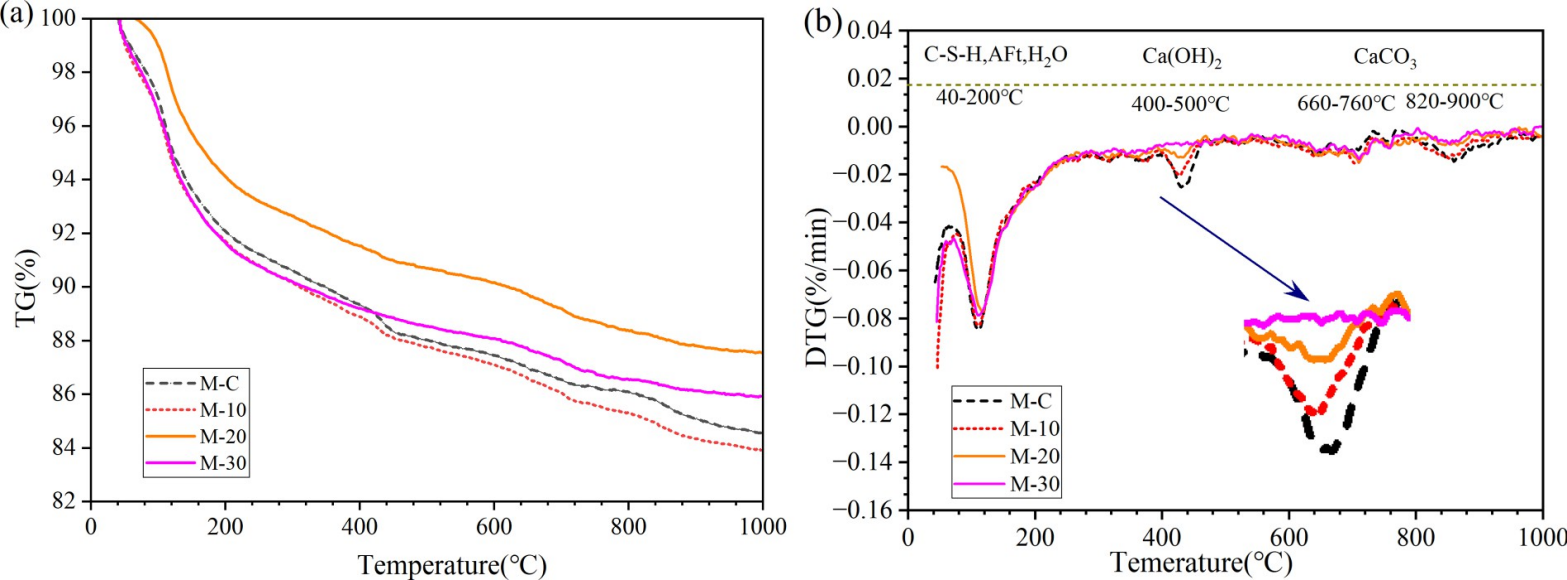
Results of analytical investigation of UHP-ECCs: (a) TG and (b) DTG curve.

Fig 7A depicts the TG curves of UHS-ECCs at 28 days, where DTG curves were obtained by the first cut linter derivative of TG curves, as shown in Fig 7B. It could be seen that four prominent peaks were presented in Fig 7B, where the first peak from 40°C to 200°C is gradually related to the vaporization of free water in a mortar and the dehydration process of C-S-H gel, AFm, and ettringite; the second peak from 400°C to 500°C is the dehydroxylation process of portlandite; the third and fourth peak is the decarbonation period of carbonate at 660°C–760°C and 820°C–900°C [33, 34].

**Fig 8:**
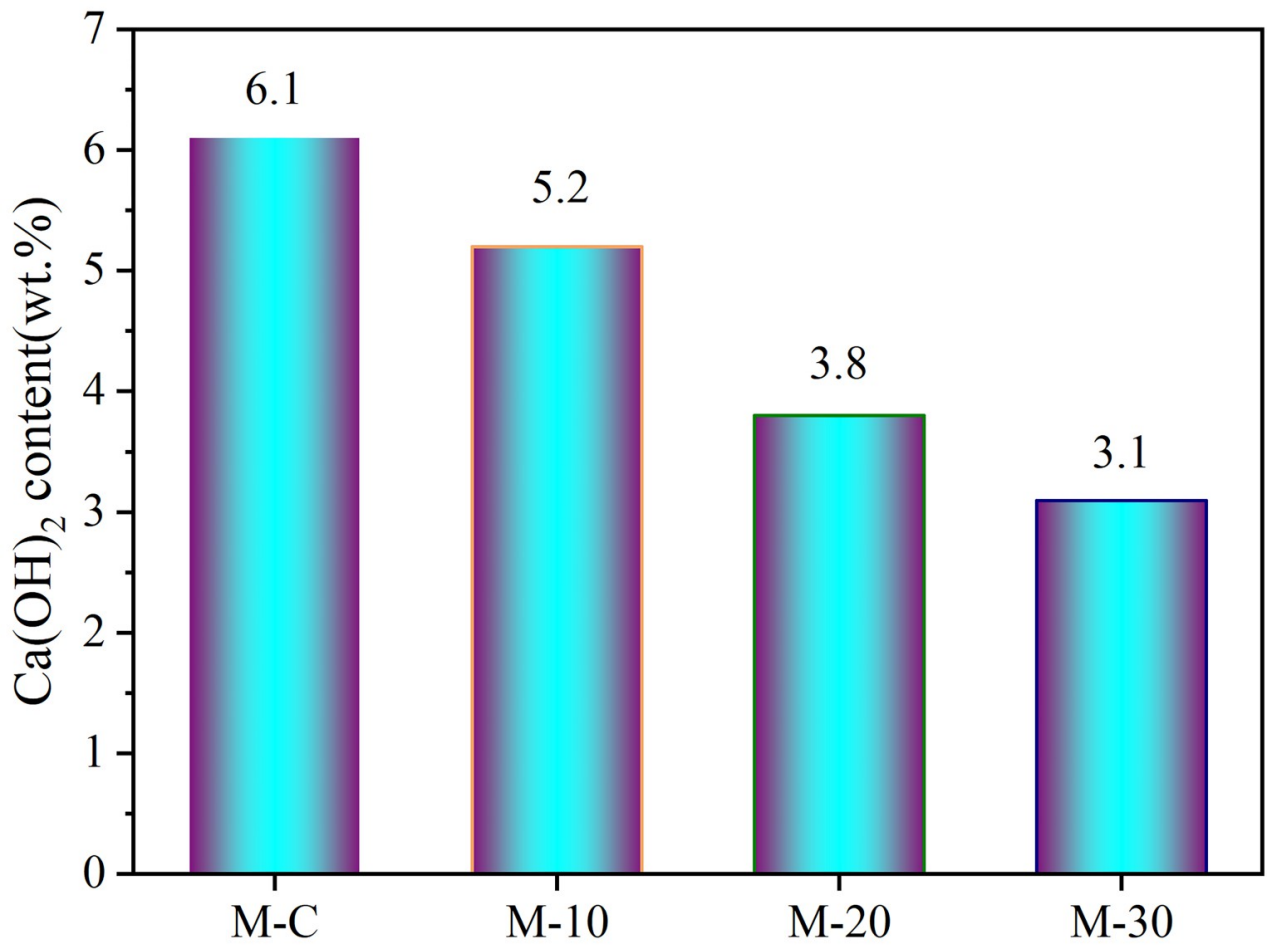
Portlandite contents in UHS-ECCs paste at 28 days.

From Fig 7B, the weight loss of portlandite in the second peak of M-C was greater than that of the other three mixtures, while the specific portlandite content of four mortars was calculated by Eq (1), as illustrated in Fig 8. As RHA dosage rises, the portlandite gradually decreases, which indicates that the addition of pozzolanic RHA consumes more portlandite due to the pozzolanic reaction. M-C has a higher portlandite which may be attributed to the maximum cement dosage. Hence, the compressive strength of M-10 and M-20 is greater than that of M-C. However, the reduction of cement also decreases the portlandite content in mortar, indicating that the substitution of cement by 30% RHA (by mass) produces less portlandite and hydration products for M-30, thus decreasing the mortar strength.

### 3.5 Tensile performance

**Fig 9:**
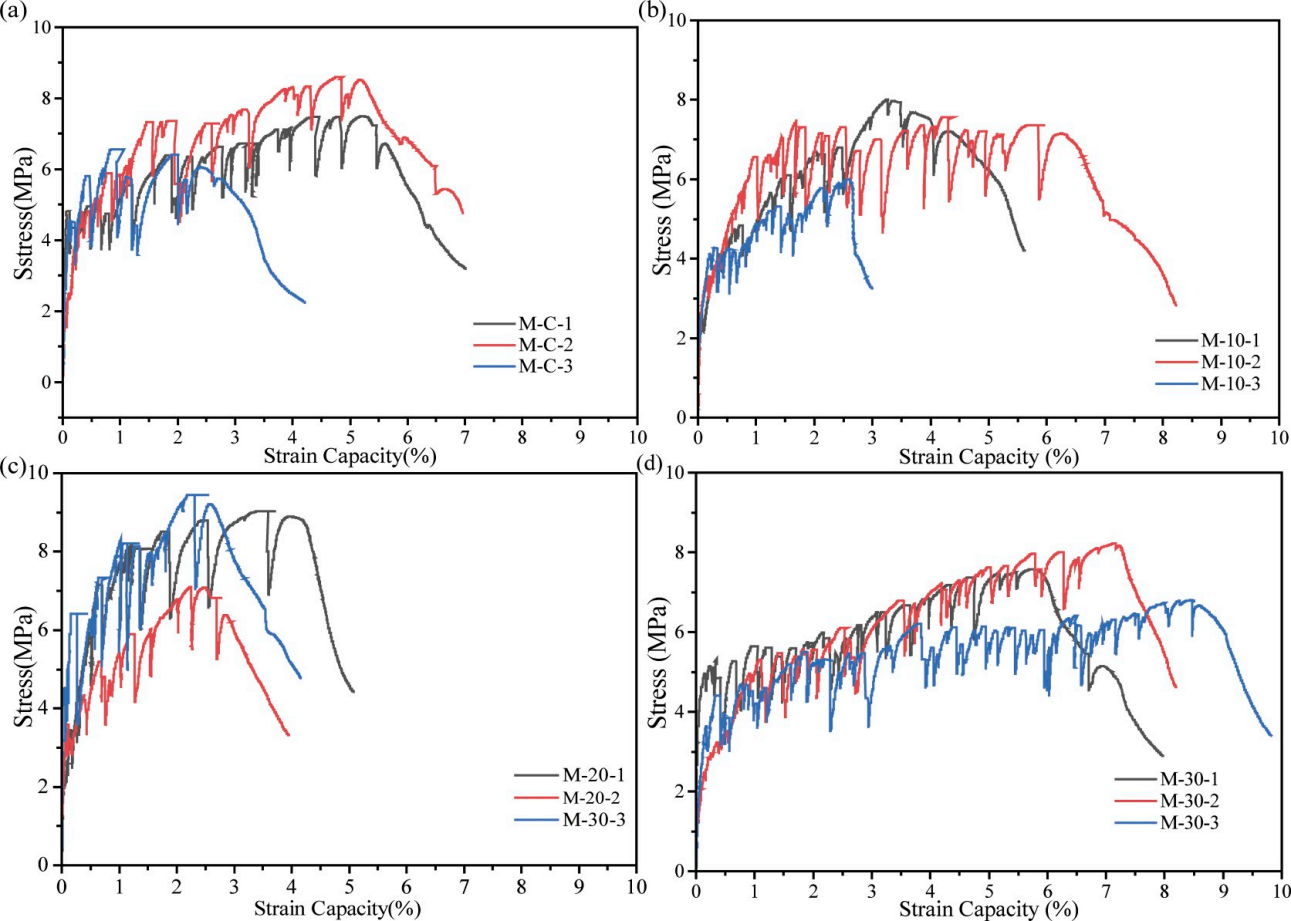
Tensile stress-strain curves of UHS-ECCs.

**Fig** 9 plots the tensile curves of UHS-ECC mixtures. Four main tensile properties containing first crack stress, peak strength, tensile ductility, and strain energy were averaged from the three specimens and depicted in Fig 11.

In this study, the first crack strength was recognized if the first strength relaxation occurred in the tensile curves. Generally, the first crack strength correlates positively with the matrix strength. As shown in Fig 11A, for four UHS-ECC mixtures, the first crack strength tends to increase and then reduce. However, the first crack strength of M-10 is near M-R, which is due to the proximal matrix strength of M-R and M-10. Overall, the first cracking strength of four UHS-ECC ranges from 3.9 MPa in M-30 to 4.7 MPa in M-20. However, the higher first crack stress could reduce the pseudo strain hardening strength (PSHstrength) index of UHS-ECC, which is unfavorable to the multiple cracking since high external energy is needed to evolve a new crack.

Regarding to the peak tensile stress of UHS-ECCs, as depicted in Fig 11B. Blending pozzolanic RHA firstly induces the increasing tensile stress of UHS-ECCs that increases from 7.54 MPa of M-R to 8.50 MPa of M-20. The tensile strength of M-10 and M-20 was enhanced by 3.1% and 12.7%, respectively, compared to that of M-R. However, M-30 with less cement content still has a tensile stress of 7.4 MPa. Overall, the tensile stress of four UHS-ECCs is greater than 5.04 MPa of C90 concrete according to Eurocode 2 [43], while the maximum recrement of tensile stress was presented in M-20 mortar.

Ultimate tensile strain, the most vital parameter, the train capacity of UHS-ECC, was impaired by incorporating RHA, especially for M-20 which only has a strain capacity of 3.2%, a remarkable decrease by 45.7% compared to that of 5.9% in M-R. For the M-10 mixture, the strain capacity is 5.3% which was decreased by 10% compared to M-R. However, the strain capacity of M-30 approaches 7.08% which is the highest train capacity in UHS-ECCs with RHA. Considering the usage of PE fiber, the slip hardening coefficient and chemical bond stress in PE fiber/matrix were near zeros, and the matrix/fiber frictional stress was mainly affected by the silica sand [44]. Hence, the decrement of tensile strain could be ascribed to the variation of fracture toughness (*K*_*m*_) which was predominantly affected by the matrix strength, which will be discussed in section 3.5. Based on the above results, a suitable RHA content should be considered to maintain the tensile ductility of UHS-ECCs.

**Fig 10:**
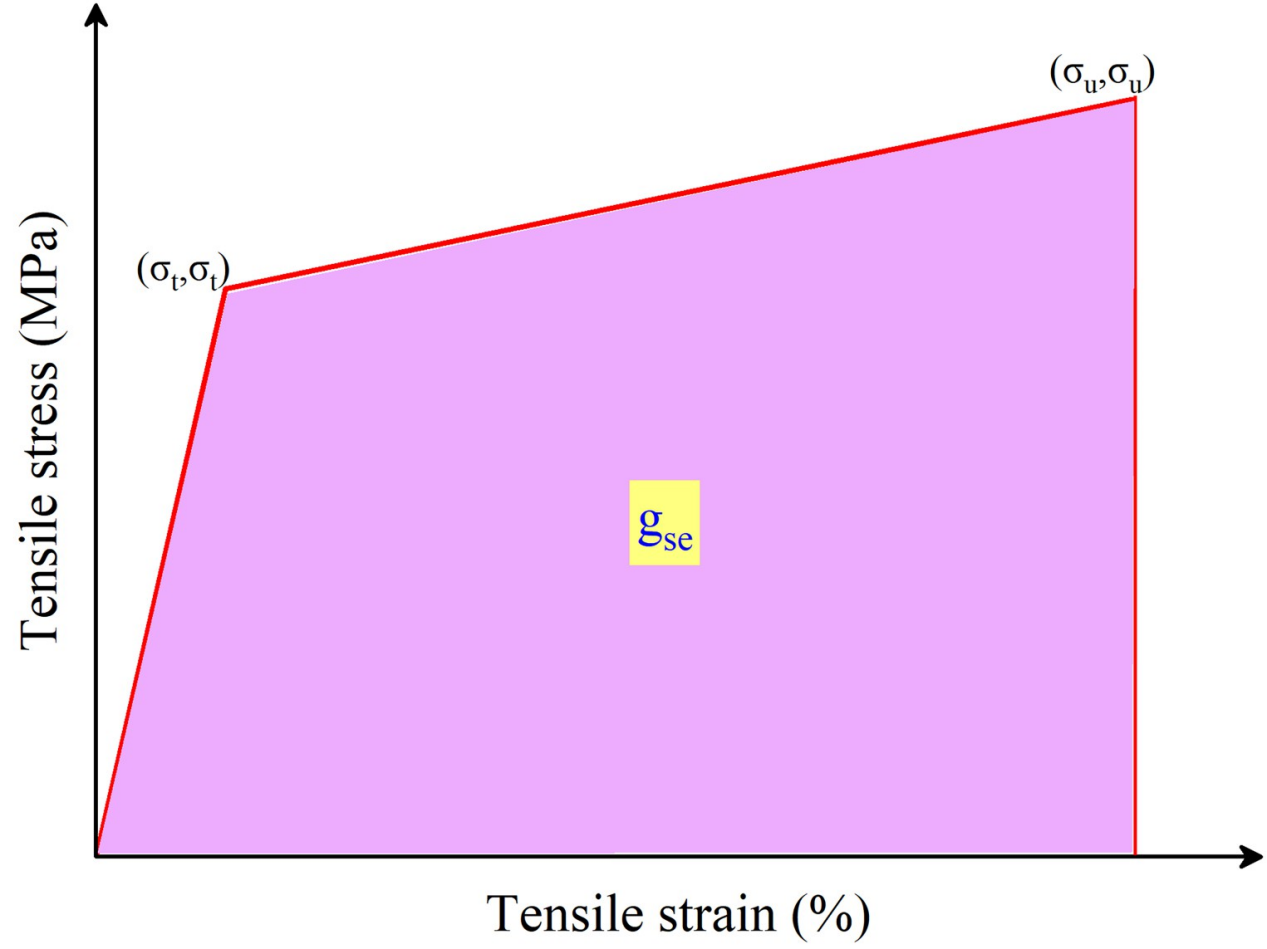
Typical tensile stress-strain relation of ECCs.

**Fig 11:**
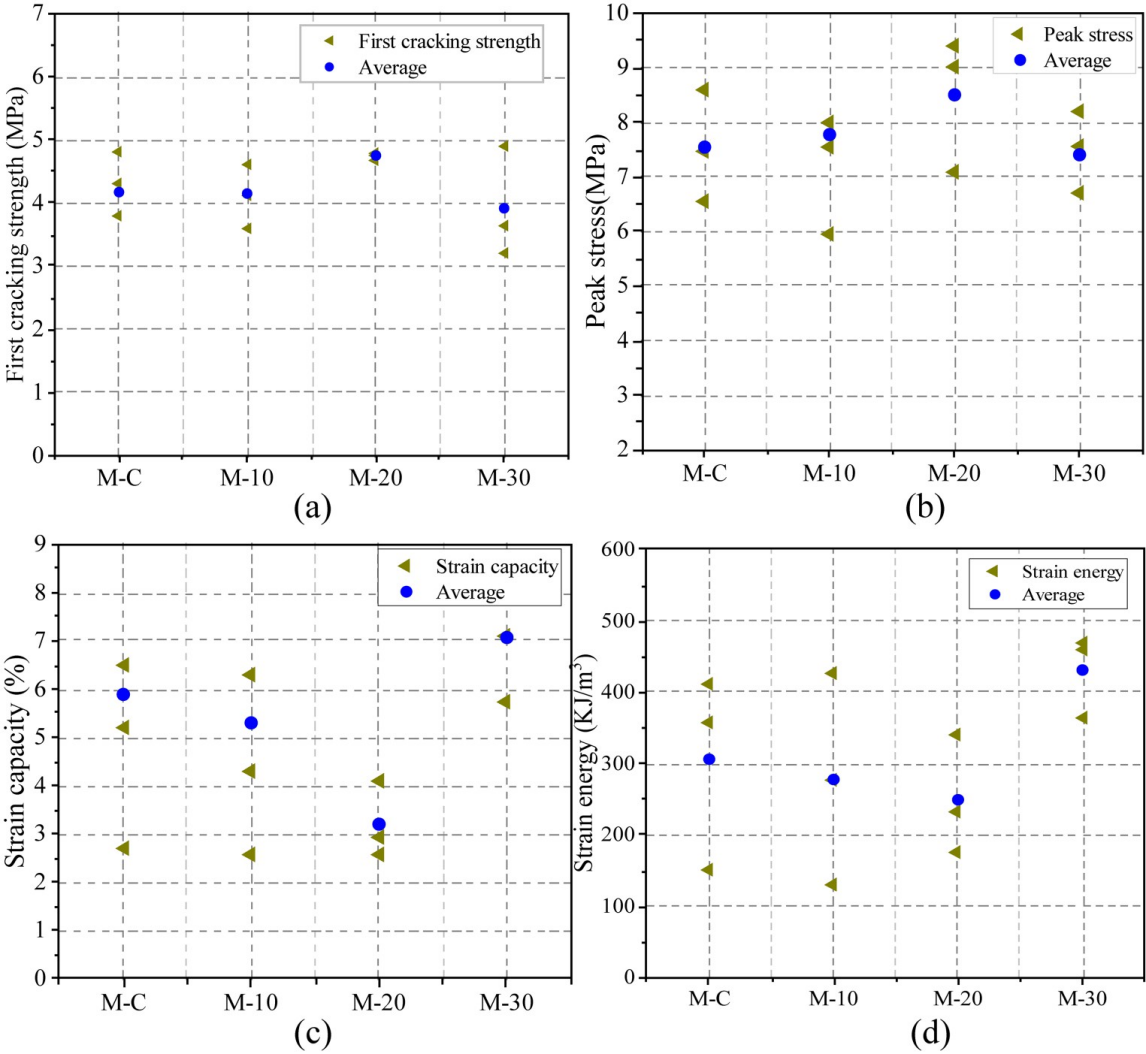
Tensile properties of UHS-ECCs; (a) first cracking strength, (b) ultimate tensile stress, (c) tensile ductility, and (d) strain energy.

Fig 10 shows the typical tensile stress-strain relation of conventional ECCs. From Fig 11C and 11D, the decreased strain capacity of M-10 and M-20 also leads to a reduced strain energy of UHS-ECCs. The mean strain energy presents the same trend as the tensile ductility and compressive strength of UHS-ECCs, which decreased from 306 KJ/m^3^ of M-R to 249 KJ/m^3^ of M-20 meanwhile M-30 has a higher strain energy of 431 KJ/m^3^. The high strain energy of all UHS-ECCs is higher than that of PVA-ECC due to the high tensile strength and ductility in PE-ECC, which is favorable to motivate the application of UHS-ECCs in seismic resistance structure.

**Fig 12:**
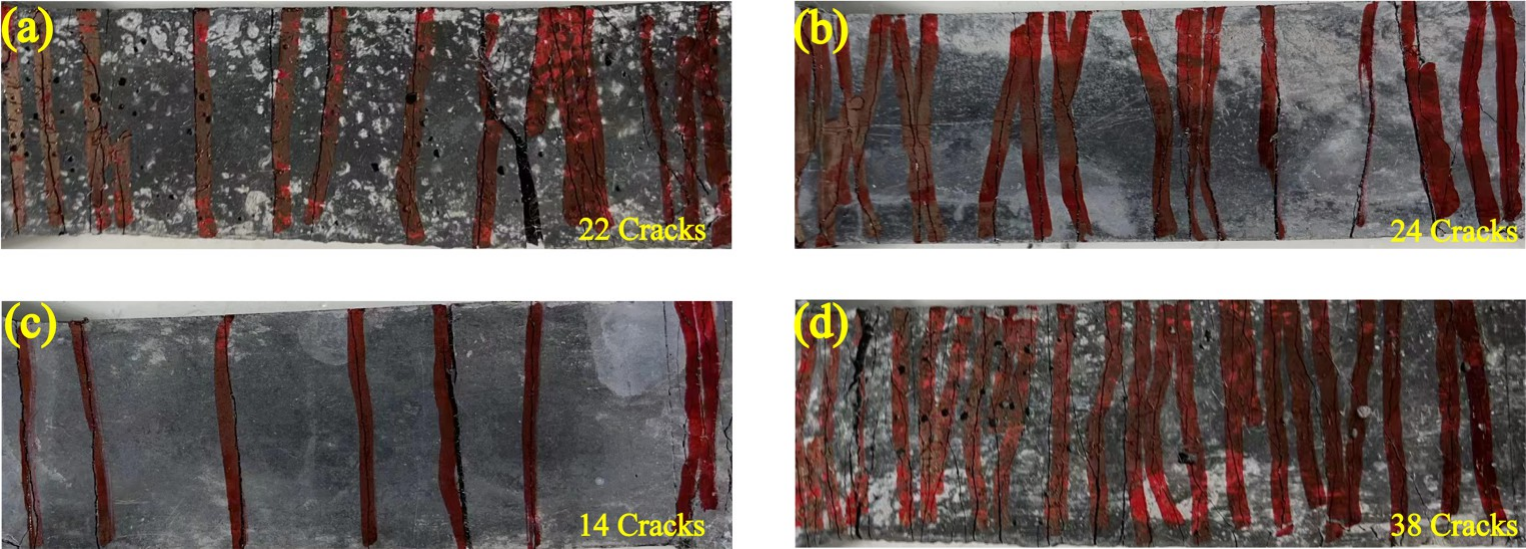
Cracking characteristic of UHS-ECCs; (a) M-C, (b)M-10, (c) M-20, and (d) M-30.

Besides, the crack numbers on UHS-ECCs are presented in Fig 12. In general, more cracks on the specimen surface indicate that saturated cracking status is easy to be launched; however, UHS-ECCs have fewer crack numbers than the other ECCs with popular high strength or popular strength [45, 46]. The M-30 possesses a higher crack number than other specimens, which could achieve a saturated cracking state accompanied by a higher tensile ductility.

### 3.6 High ductility analysis of UHS-ECC

The significant strain-hardening properties including pseudo strain-hardening strength (*PSH strength*) and pseudo strain-hardening energy (*PSH energy*) were calculated to interpret the effect of RHA content on the ductility of UHS-ECCs mixtures. On the basis of criteria energy and strength criteria of the traditional ECC [47], the fiber bridging complementary energy *J*_*b*_′ and crack tip toughness *J*_*tip*_ are the main key parameters to impact cracks opening process, thus influencing the ductility of UHS-ECCs. In summary, a lower matrix strength has more flaws, which is usual to possess lower fracture toughness. Meanwhile, a higher *J*_*b*_′ with a low *J*_*tip*_ would effectively promote the multiply cracks opening; thus, the significant index of *PSHEnergy* was conducted to analyze the tensile ductility of ECC per Eq (4).

**Fig 13:**
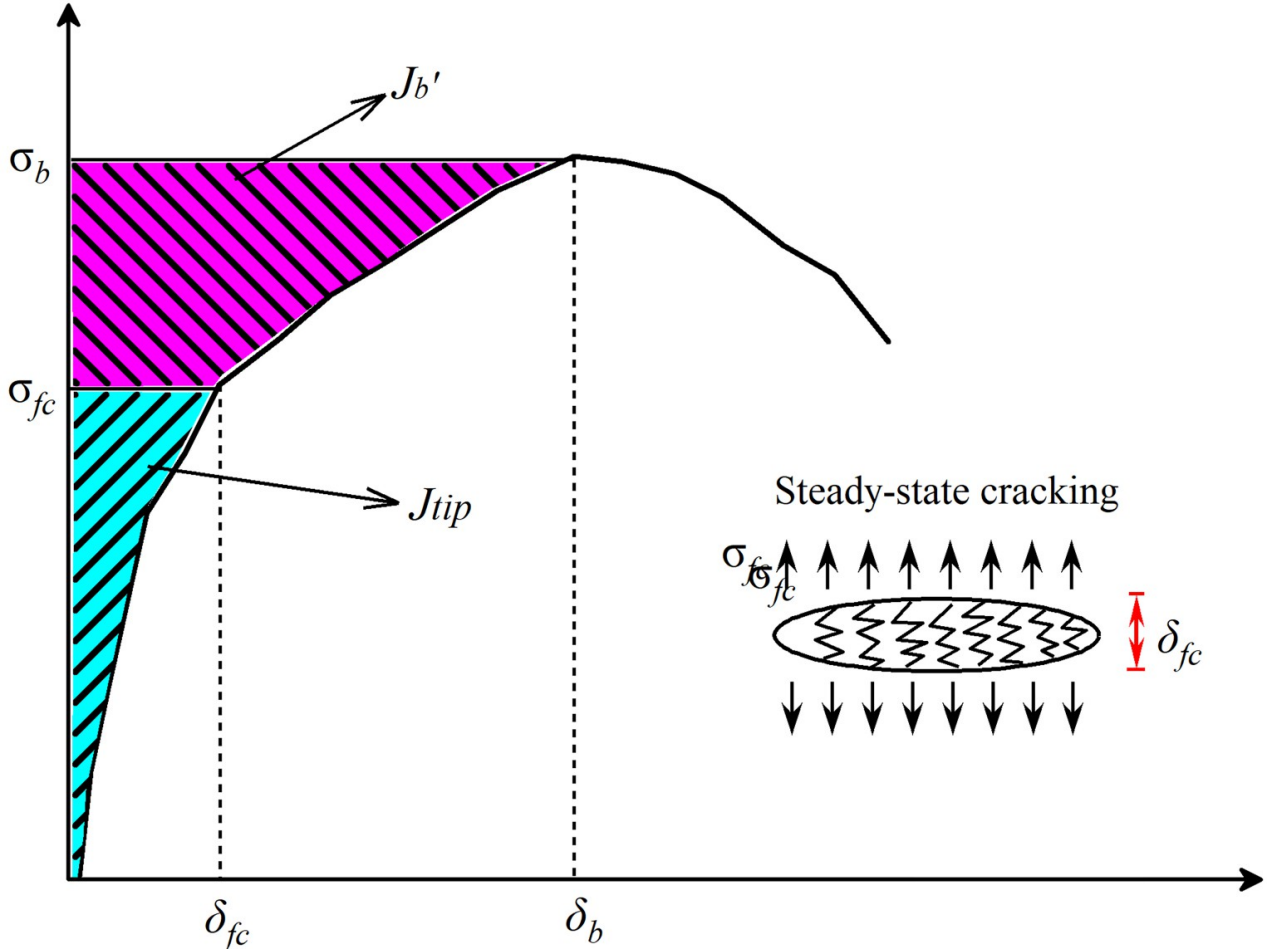
Typical σ-δ relation for engineered cementitious composites.

Fig 13 plots the typical σ-δ relation for UHS-ECC. The *J*_*b*_′ and *J*_*tip*_ could be derived by Eqs (2)–(7). *E*_*m*_ is the tensile elastic modulus of UHS-ECCs; however, it was difficult to be accurately gained due to the limitation of measure equipment; thus, Eq (4) was employed to capture the *E*_*m*_ of matrix [48]. *σ*_*b*_ and *δ*_*b*_ represent the peak stress and homologous crack opening width, respectively. *σ*_*fc*_ and *δ*_*fc*_ represent the peak stress and homologous crack opening width during the stable tensile process, respectively. *K*_*m*_ is the matrix fracture toughness, which could be obtained by Eq (5) according to ASTM E399 [37]. Moreover, in Eq (5), *P*_*s*_ is the maximum load of the three-point flexure test; *S* is the span of the sample; a is the height of the prefabricated crack; *B* is the thickness; *W* is the weight of the specimens. In Eq (7), *σ*_*c*_ is the peak tensile strength in the classical σ-δ curve for ECC, while *σ*_0_ is the first crack tensile strength in Fig 9A [47].

**Fig 14:**
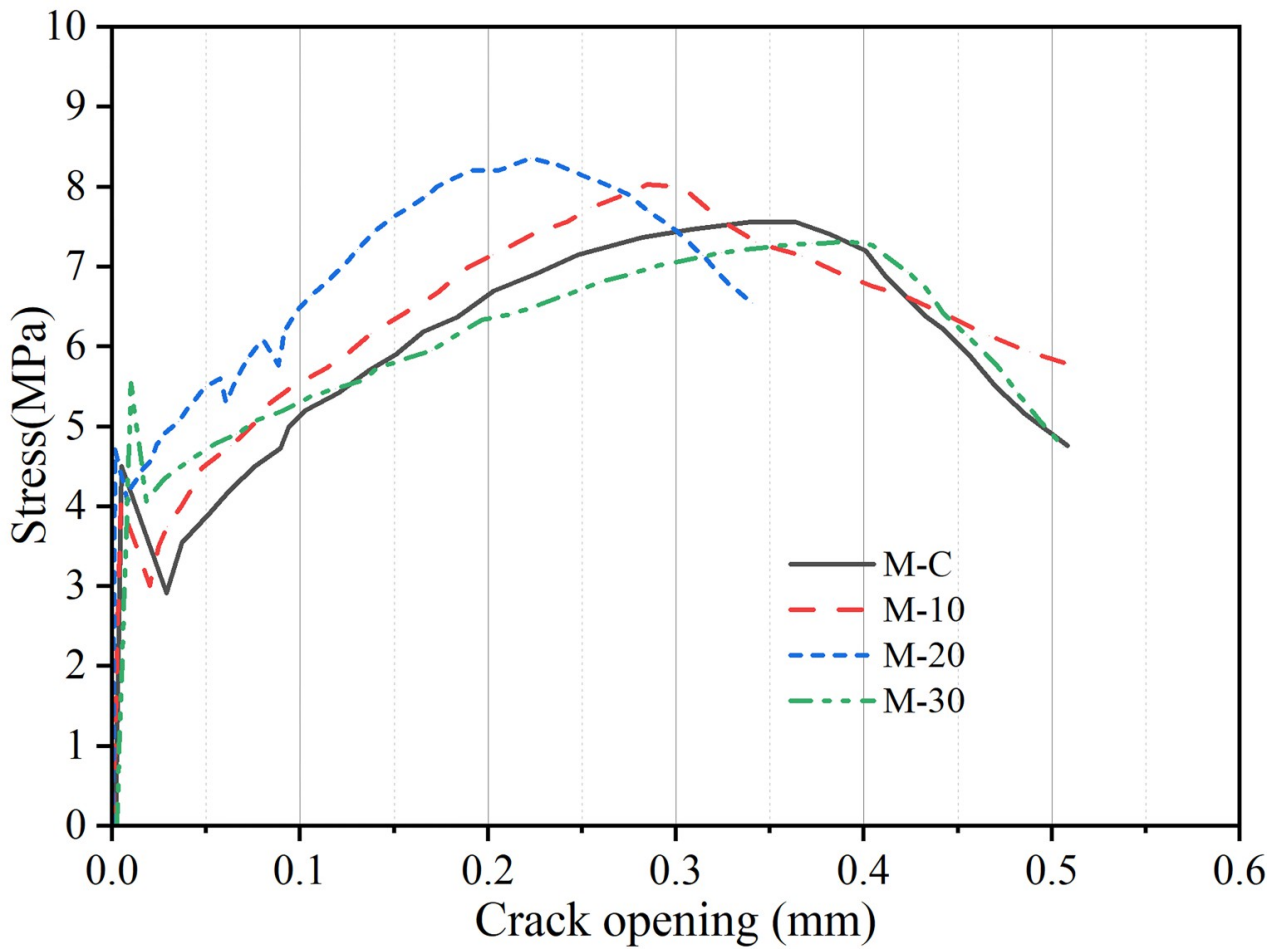
Fiber bridging stress-crack opening width relationships of UHS-ECCs.

Fig 14 depicts the fiber bridging stress-crack opening width relationships of UHS-ECCs. According to Eqs (2)–(7) [13], the significant parameter of *PSHenergy* and *PSHstrength* can be calculated, as presented in Table 4. As the increment of RHA content, the *E*_*m*_ first enhance and then decrease, and the *K*_*m*_ presented a proximal value for M-10 and M-20, while a low *K*_*m*_ was exhibited. Moreover, the *E*_*m*_ of the matrix was slightly enhanced and decrease with RHA content increases, and these experimental results were consistent with the changing strength of UHS-ECCs. However, the *PSHstrength* and *PSHenergy* index of all UHS-ECCs are higher than the required minimum threshold of 1.2 and 3 for ECC with PE fibers, respectively [49], demonstrating that all UHS-ECCs could present a multi-cracks opening process and show a favorable pseudo strain hardening curves. M-20 has a minimum *PSHstrength* and *PSHenergy*, thus leads to a lower ductility than other three UHS-ECCs, which could mainly be ascribed to the decreasing fiber bridging complementary energy *J*_*b*_′. However, per preference Zhang [24], as the increment of black RHA content (by volume), the strain capability of high-strength ECC would increase, and the matrix strength was also enhanced, which presents a diverse conclusion with this study. Hence, for UHS-ECCs containing pozzolanic RHA in this study, the tensile ductility of UHS-ECC would gradually decrease if the substituted content of cement by pozzolanic RHA was lower than 20% (by mass).


$$J_{tip}\leq \sigma_{b}\delta_{b}-\int_{0}^{\delta_{b}}\sigma (\delta )d\delta ={J_{b}}^{\prime }$$ (2)

$$J_{tip}=\frac{{K_{m}}^{2}}{E_{m}}$$ (3)

$$E_{m}=0.83\times 2.15\times 10^{4}\times {\frac{\sigma }{10}}^{1/3}$$ (4)

$$K_{m}=\frac{P_{s}S}{BW^{3/2}}∙3\sqrt{\frac{a}{W}}∙\frac{1.99-(\frac{a}{W})(1-\frac{a}{W})[2.15-3.93\frac{a}{W}+2.7{\left(\frac{a}{W}\right)}^{2}]}{2(1+2\frac{a}{W}){\left(1-\frac{a}{W}\right)}^{\frac{3}{2}}}$$ (5)

$$PSHenergy=\frac{{J_{b}}^{\prime }}{J_{tip}}$$ (6)

$$PSHstrength=\frac{\sigma_{c}}{\sigma_{0}}$$ (7)

**Table 4:** Fracture test results and calculated PSH indices for UHP-ECC.

Number	*Em* (GPa)	*Km* (MPa· m^1/2^)	Strength Criterion			Energy Criterion		
			*σ*_*c*_ (MPa)	*σ*_*0*_ (MPa)	*PSH*_*strength*_	*J*_*tip*_ (J/m^2^)	*J*_*b*_*’* (J/m^2^)	*PSH*_*energy*_
**M-C**	38.6	0.699	7.56	4.17	1.81	12.48	484.6	38.3
**M-10**	38.7	0.686	8.03	4.15	1.93	12.0	432.8	36.0
**M-20**	39.2	0.70	8.35	4.75	1.76	12.60	390.9	31.0
**M-30**	37.4	0.60	7.26	3.91	1.86	9.61	587	61.0

### 3.7 Microstructure of UHS-ECC

**Fig 15:**
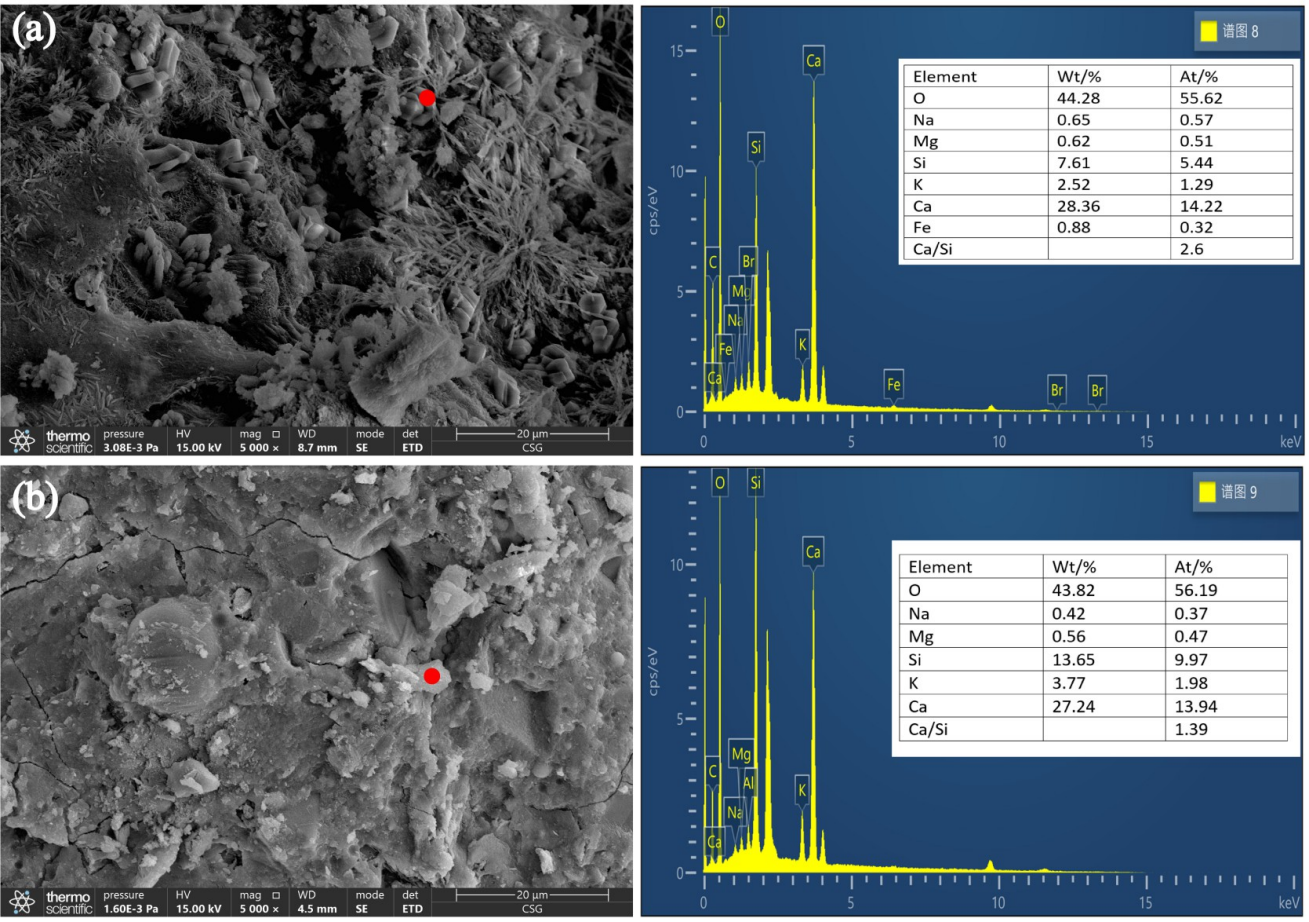
SEM images of UHS-ECCs. (a) M-C; (b) M-20.

Fig 15 shows the SEM images of UHS-ECCs. From Fig 15(A), it could be observed that amount of needle-like ettringites in the microstructure of the matrix, while the flaky portlandites were obviously seen in M-R, indicating that many portlandites have not been consumed in M-R. In addition, it can be seen that remote portlandite and ettringites are presented in M-20, which was likely due to the pozzolanic effect of RHA that react with Ca(OH)_2_ to manufacture new C-S-H gel [50], and the physical filling effect of RHA that dense the interfacial transition (ITZ) zone between sand and matrix, thus leading to a compacted microstructure of M-20. On the other hand, from the EDX-mapping analysis results, the Ca/Si of C-S-H gel in M-20 is 1.39 which is lower than that of M-R, which demonstrates that massive C-S-H gel with a low Ca/Si is produced during the pozzolanic reaction of RHA, thus contributing the gaining strength of M-20. Hence, incorporating RHA was favorable to dense the microstructure and enhance the matrix strength.

## 4. Conclusions

In this paper, the impact of pozzolanic RHA on the workability and mechanical properties including the compressive strength and tensile properties of ultra-high strength ECCs were studied systematically. In addition, the composition pattern and quantity of hydration products were analyzed by XRD and TG analyses. Moreover, the *PSH* index for all UHS-ECCs was deduced to interpret the ductility of UHS-ECCs with varying RHA dosages. Finally, the morphology and element composition of UHS-ECCs with RHA were observed by SEM analysis accompanied by an EDS detector. All the findings of this study are as follows:

1. As the RHA dosage increases, the fluidity of UHS-ECCs mortar reduces from 205mm to 165mm due to a large specific surface area of pozzolanic RHA that exhibits high absorbing water property. Moreover, the lubrication effect was not represented for RHA since ground RHA has an irregular angular structure that increased the friction force between the flowing particles. Hence, to ensure the workability of UHS-ECCs, the replacement of RHA should be lower than 20% of cement (by mass).
2. The compressive strength of UHS-ECCS presents a first increasing tendency and then reduces with the increment of pozzolanic RHA content, which ranges from 125.8 MPa to 130.8 MPa due to its substantial amorphous silica that shows a pozzolanic effect which was captured by XRD analysis and TG analysis results. Moreover, the filling effect of RHA also could enhance the ITZ properties of the matrix, thus improving the mortar strength;
3. The tensile stress and first crack tensile strength of UHS-ECCs enhance first and then decrease, which could achieve a maximum tensile strength of 8.5 MPa which is two times of popular C90/C105 concrete. Moreover, the tensile capacity and tensile energy of UHS-ECCs present a decreasing tendency and then increase, which could reach the highest tensile capacity of 7.08%. However, when the substituted cement of cement by RHA was lower than 20%, blending RHA would decrease the ductility due to the decrement of fiber bridging complementary energy, which was unfavorable to the cracking process.
4. At microstructure level, incorporating RHA was favorable to dense the microstructure due to its chemical properties that the portlandite in a mortar containing RHA would react with RHA to produce new C-S-H gel. The amount of C-S-H gel with a low Ca/Si is formed during the pozzolanic reaction of RHA, which has a higher strength than the popular C-SH gel with a Ca/Si of over 1.5, thus contributing to the gaining strength of M-20.
5. Overall, a utril-high strength ECC with a compressive strength of 130.8 MPa, a ductility of 3.2%, and a serviceable fluidity of 182 mm was developed. To ensure the UHS-ECCs with the comprehensive characteristics of ultra-high strength, high tensile ductility, and serviceable workability, the replacement of pozzolanic RHA should not exceed 20% of cement by mass.

## 5. Outlook

Encouragingly, considering the environmental problems related to the disposal of RHA, the use of pozzolanic RHA to prepare the UHS-ECC is an important matter, which contributes to providing a clear motive for the sustainable development of UHS-ECC. However, to fulfill the comprehensive research of pozzolanic RHA in UHS-ECC, some significant work still needs to be implemented in future as follows:

1. RHA used in this work has great pozzolanic activity due to its massive amorphous silica under 700°C, indicating that the industrial mass production technology of RHA should be researched.
2. RHA possesses a large specific surface area, leading to superior hydrophilia that also could absorb the superplasticizer. Hence, a special superplasticizer should be designed to enhance the dispersivity of particles in paste containing pozzolanic RHA. Moreover, the effect of RHA on the mortar rheology should be further investigated to obtain the optimal plastic viscosity which makes fibers homogeneous disperses.
3. At micro-scale, the influence of RHA on the micromechanics including frictional bonding strength and slip-hardening coefficient could be further explored by a single fiber pull-out test.
4. The internal curing effect of RHA on the comprehensive properties at late age is worthy to be further explored to ensure the strength and durability of UHS-ECC.
